# The Institutional Library

**Published:** 1921-05-14

**Authors:** 


### THE INSTITUTIONAL LIBRARY.

**The Right to Strike.** By Ernest Hutchinson and George Goodchild. (Hayes. Price 2s. 6d. net.)

This novel, "written up" from a recent successful play dealing with the ethics of strikes, suffers from the usual defects of construction attending this method of composition; and to literary form and style it has no pretensions. The central theme is the moral justification of strikes by large bodies of specialised workers entered upon with express intent to coerce the community by depriving it of some vital amenity of civilised existence. In the story a large urban population served by a single line of railway, and unable to continue its economic prosperity—even to get the food necessary for subsistence—is "held up" by a railway strike. The strike leader connives at the murder, by extremists among his followers, of a local doctor who is helping to feed the town by a motor-lorry service; whereupon all the doctors in the district refuse attendance upon strikers and their dependants. A child dies of undiagnosed diphtheria, and this afflicts the conscience of one of the doctors; and when the strike leader's wife is stricken with disease requiring immediate operation this doctor and a surgeon relent at the last moment—after the latter has been "prohibited from practising" by (of all people) the solicitor to the British Medical Association!! At the same time, the strike has been settled behind the back of the extremist leaders by a half-way house compromise between the company and the workers. The authors may argue that they are not propagandists, either for socialism, bolshevism, or for established institutions; and therefore that it is their aim to suggest points for reflection rather than to take sides. All the same, they have propounded a question and then shirked answering it.

**Blood Pictures: An Introduction to Clinical Hæmatology.** By Cecil Price-Jones, M.B. (Lond.). Second edition. (London: John Wright & Sons, Ltd., and Simpkin, Marshall, Hamilton, Kent & Co., Ltd. Price 6s. 6d. net.)

The second edition of this little volume does not greatly differ from the first. Some minor errors have been corrected, and one is glad to see an illustrated description of the improved Bürker's hæmocytometer, the older forms of instrument being now unprocurable in this country. The author's aim is to provide a guide to the straightforward clinical interpretation of blood examination reports, and, without dealing with the theoretical aspect of the subject, Mr. Price-Jones has achieved considerable success. The binding of the present edition is not so pleasing as the original, but the revised text retains all its essential value.

**Heart Disease: Graphic Methods.** By John Hay, M.D., F.R.C.P. Pp. 178. (Oxford Medical Publications, Henry Frowde, Hodder & Stoughton. Second edition. 12s. 6d. net.)

It is eleven years since the appearance of the first edition, and during this time many important advances have been made in cardiology. Although medicine is not one of the exact sciences, it is the aim of every teacher to train his students to become careful, competent, and reasoning observers. The introduction of graphic methods makes the student's task easier as, in most cases, these methods give him a very fair representation of the physiological state of the organ he is studying: they also tend to eliminate the personal element, which is very desirable in the securing of scientific data.

This little book gives an excellent account of the uses of the polygraph, an instrument which every student should master whilst in hospital and continue to use on his cardiac cases in practice. Useful tracings of arterial and venous pulses are also given.

**Care and Feeding of Infants and Children.** By W. R. Ramsey, M.D. Second Edition. Pp. 290. With 123 illustrations. (Philadelphia and London: J. B. Lippincott Co. 1920. Price 10s. 6d.)

Dr. W. R. Ramsey, in the second edition of his textbook on *The Care and Feeding of Infants and Children*, gives us a work which is peculiarly American in both conception and technique. The author advocates the supervision of children from birth to school age by specially-trained Public-Health nurses, the registration of pregnancies at an early stage and the importance of hygienic pre-natal surroundings. The chapters devoted to anatomy and physiology and the growth and development of the child are interesting and instructive, and the charts giving the weight and height of the average child up to the sixteenth year are of great practical value.

Dr. Ramsey has made an exhaustive study of the caloric values of simple foods in their relation to the artificial feeding of infants, and his arguments in favour of a wider use of the butter-milk formula are sound. The book is admirably illustrated and contains a list of simple foods and their caloric values.

**Model Answers to Questions on Nursing, set by the Medico-Psychological Association.** By Hector Macphail. (London: The Scientific Press, Ltd., 28-29 Southampton Street, Strand, London, W.C. 2. Third edition. Price 6s. net.)

In its third edition this work appears in fully revised and slightly enlarged form. It should prove of the greatest assistance to candidates for the nursing certificate of the Medico-Psychological Association of Great Britain and Ireland, and the great lesson which it carries is that questions must be answered briefly yet clearly and to the point. As an aid to success in study, and particularly in the examination itself, candidates will find this little book uncommonly hard to beat.

**Bio-Moulding Origins in Evolution.** By Dr. H. Elliot-Blake. Publisher's name not given.

This book consists of a series of essays couched in semi-scientific jargon and plentifully interlarded with passages in italics. With the best will in the world to find a meaning or a coherent line of reasoning in the book, the present writer has completely failed in doing so.

**First-Aid in Emergencies.** By Eldridge L. Eliason, A.B., M.D., F.A.C.S. (Philadelphia and London: J. B. Lippincott Co. 7s. 6d. net. Third edition revised 1920.)

This well-known manual, first published in 1915, has been considerably improved. In particular the method of fixation and dressings of fractures found to be best in the war is fully described and illustrated. The illustrations and numerous diagrams with which the work is interspersed are admirably calculated to give a clear impression to the learner. It is compact in size, well printed, and well adapted for its purpose.

